# Virtual Screening of Different Subclasses of Lignans with Anticancer Potential and Based on Genetic Profile

**DOI:** 10.3390/molecules28166011

**Published:** 2023-08-11

**Authors:** Mayara dos Santos Maia, Francisco Jaime Bezerra Mendonça-Junior, Gabriela Cristina Soares Rodrigues, Adriano Soares da Silva, Niara Isis Pereira de Oliveira, Pablo Rayff da Silva, Cícero Francisco Bezerra Felipe, Ana Pavla Almeida Diniz Gurgel, Anuraj Nayarisseri, Marcus Tullius Scotti, Luciana Scotti

**Affiliations:** 1 Department of Molecular Biology, Federal University of Paraíba, João Pessoa 58051-900, PB, Brazil; mayarasm@ltf.ufpb.br; 2 Laboratory of Synthesis and Drug Delivery, State Universtiy of Paraiba, João Pessoa 58071-160, PB, Brazil; 3 Postgraduate Program in Natural Synthetic and Bioactive Products (PgPNSB), Federal University of Paraíba, João Pessoa 58033-455, PB, Brazil; pablo-rayff@hotmail.com (P.R.d.S.); cicero@dbm.ufpb.br (C.F.B.F.); luciana.scotti@gmail.com (M.T.S.); mtscotti@gmail.com (L.S.); 4 Miriri Foods and Bioenergy S/A, Santa Rita 58300-970, PB, Brazil; gaby.ecologia@gmail.com; 5 Program in Ecology and Environmental Monitoring, Federal University of Paraíba, João Pessoa 58059-900, PB, Brazil; adriano.soares05071997@gmail.com (A.S.d.S.); naira.isis.ni@gmail.com (N.I.P.d.O.); 6 Program in Cellular and Molecular Biology, Federal University of Paraíba, João Pessoa 58051-900, PB, Brazil; ana.pavla@academico.ufpb.br; 7 In Silico Research Laboratory, Eminent Bioscience, Indore 452010, Madhya Pradesh, India; anuraj@eminentbio.com; 8 Laboratory of Cheminformatics, Health Sciences Center, Federal University of Paraíba, João Pessoa 58033-455, PB, Brazil

**Keywords:** lignans, cancer, single nucleotide polymorphisms, virtual screening

## Abstract

Cancer is a multifactorial disease that continues to increase. Lignans are known to be important anticancer agents. However, due to the structural diversity of lignans, it is difficult to associate anticancer activity with a particular subclass. Therefore, the present study sought to evaluate the association of lignan subclasses with antitumor activity, considering the genetic profile of the variants of the selected targets. To do so, predictive models were built against the targets tyrosine-protein kinase ABL (ABL), epidermal growth factor receptor erbB1 (EGFR), histone deacetylase (HDAC), serine/threonine-protein kinase mTOR (mTOR) and poly [ADP-ribose] polymerase-1 (PARP1). Then, single nucleotide polymorphisms were mapped, target mutations were designed, and molecular docking was performed with the lignans with the best predicted biological activity. The results showed more anticancer activity in the dibenzocyclooctadiene, furofuran and aryltetralin subclasses. The lignans with the best predictive values of biological activity showed varying binding energy results in the presence of certain genetic variants.

## 1. Introduction

The incidence of many types of cancer continues to rise in the global population, despite many successes in screening, prevention and treatment [1]. Several factors contribute to the development and progression of cancer, such as resistance to various drugs and mutations in important genes [2]. The sum of changes in oncogenes, tumor suppressor genes, repair mechanisms and epigenetic changes has led to cancer development [3]. It is impossible to infer how many independent events are required to produce all the changes that result in most human cancers [4]. Therefore, continuous discovery of new therapeutic alternatives is necessary.

In addition, 40% of the interindividual differences are responsible for the varying response to treatment of patients with the same type of cancer. Genetic factors such as single nucleotide polymorphisms (SNPs) are reported as important variants that may affect treatment [5,6]. Furthermore, natural human genetic variation can cause individuals to respond differently to the same drug [7]. Thus, it is also necessary to identify new therapeutic alternatives based on the genetic profile of the patient.

Several studies have focused on enzymes as important therapeutic targets for various types of tumors. Tyrosine kinases belonging to the ABL family act in coordinating and remodeling actin in response to stimuli mediated by tyrosine phosphorylation of actin cytoskeletal remodeling proteins and by the adhesion and aggregation domain of carboxy-terminal filamentous actin ABL [8]. The ABL family performs a variety of activities which are vital to cell maintenance, such as division, adhesion to membranes, cellular skeletal remodeling and DNA damage repair [9]. However, mutations or abnormalities in tyrosine kinase activity can result in an uninterrupted disruption of the highly active state that can lead to cancer development and progression [10].

The epidermal growth factor receptor (EGFR) acts as a transcription factor that plays a regulatory role in the expression of many genes important for inflammation [11]. EGFR belongs to the ErbR family, which can irregularly activate epithelial tumors [11]. Histone deacetylases (HDACs) are key enzymes that have a regulatory function of gene expression related to controlling cell cycle advancement and apoptosis, boosting tumorigenesis and favoring the evolution of cancer [12,13,14]. mTOR threonine-protein kinase (mTOR) is an enzyme formed by two structural complexes, namely, mammalian rapamycin complex 1 (mammalian target of rapamycin complex 1) (mTORC1) and mammalian target of rapamycin complex 2 (mammalian target of rapamycin complex 2) (mTORC2). mTORC1 performs the function of regulating cell growth and metabolism, while mTORC2 controls cell proliferation and survival [15,16,17]. mTOR plays a functional role in tumor formation and is widely used in targeted therapy research for tumors and other diseases [18].

The polyadenosine diphosphate ribose polymerase (PARP) superfamily is made up of proteins and enzymes that are responsible for regulating the identification and repair process of deoxyribonucleic acid molecules through the BER pathway [19]. PARP1 is the best known and acts as a catalyst for ADP-ribose units of the NAD+ substrate in nuclear proteins. This process is performed as a post-translational modification necessary for activating the response to DNA damage generated by ionizing radiation, alkylating agents and/or free radicals [20]. PARP inhibitors destabilize replication forks through entrapment of PARP DNA and induce cell death through replication stress-induced mitotic catastrophe [21].

Targeted therapy is usually the initial treatment for a cancer patient. More than 60% of antitumor drugs are closely related to natural products [10]. Lignans are natural products made up of phenylpropanoid dimers and have a wide variety of biological activities. Many studies have reported lignans as potent anticancer agents [22,23,24]. However, as there are 10 extremely diverse subclasses of lignans, it is difficult to correlate anticancer activity with chemical structure. In addition, genetic variations cause different responses to treatment, requiring personalized treatment per patient. Therefore, the present study aims to select and evaluate the association of lignan subclasses with antitumor activity, considering the genetic profile of important variants.

## 2. Results

### 2.1. Quantitative Modeling of the Structure–Activity Relationship (QSAR)

The external performances of the selected models were analyzed for sensitivity (true positive rate or active rate), specificity (true negative rate or inactive rate) and accuracy (overall predictability). These parameters are the most used to ensure high predictability of the model. Other parameters such as ROC curve results and MCC analyzes revealed excellent results. The models obtained ROC curves greater than 0.89 during cross-validation, and MCC values were also greater than 0.63 during cross-validation, revealing a model with excellent classification, performance and robustness (Table 1, Figure S1). Thus, the lignan bank was screened using the created models with excellent performance to select potentially active compounds against the selected proteins.

**Figure 1:**
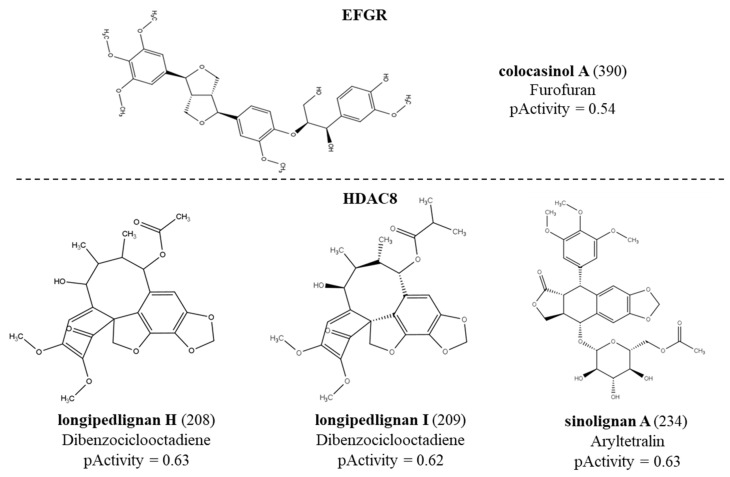

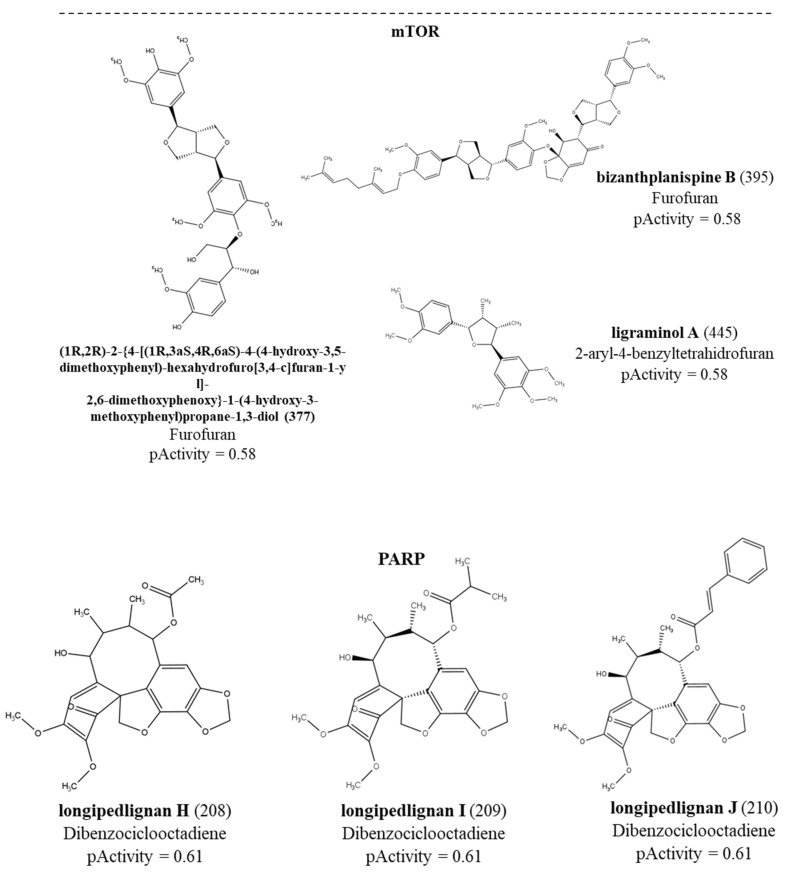
Structures of the lignans with the highest pActivity values for each protein.

**Figure 2:**
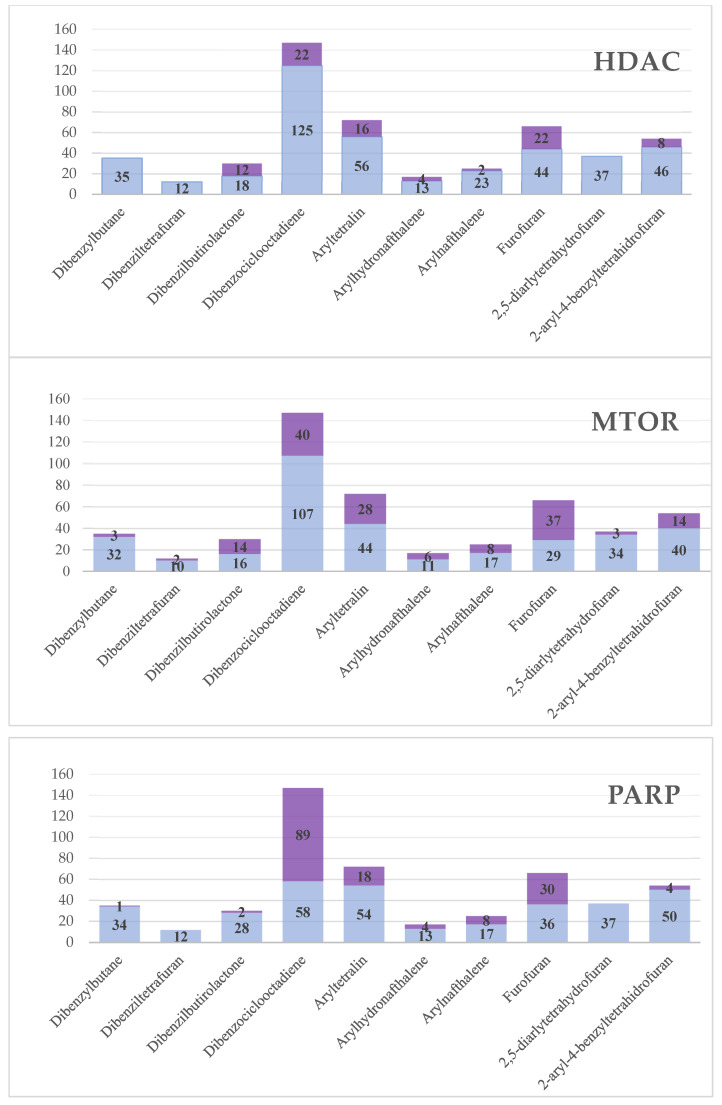
Number of lignans considered active and inactive for antitumor activity. In purple, we highlight the amount of active lignans.

The RF model was able to select a lignan with potential activity against the EFGR receptor with a probability of activity of 54%. Next, 86 lignans were considered active for the HDAC enzyme, with activity probabilities ranging from 50 to 63%. Then, 155 active compounds with activity ranging from 50 to 58% were selected for the mTOR protein. The model selected 156 compounds for the PARP enzyme with activity ranging from 50 to 56%. No lignans were active for the ABL enzyme. We noticed that the only active compound against the EFGR enzyme was colocasinol A (390). Table 2 shows details of these results by subclasses. The structure, subclass and compounds with the highest predicted biological activity values in the QSAR modeling for each enzyme can be seen in Figure 1. We observed that although the HDAC model selected fewer active compounds when compared to the mTOR and PARP models, the HDAC model was able to select compounds with greater potential for biological activity. Moreover, active compounds were seen for all subclasses for the mTOR protein. The subclass with the most active compounds for PARP was dibenzocyclooctadiene. Furthermore, we also noticed that the subclasses with more active compounds were dibenzocyclooctadiene, furofuran and aryltetralin for all proteins. Figure 2 represents the distribution of active and inactive compounds for each analyzed protein, except for ABL, which did not present an active compound, and for EFGR, which only obtained one active compound.

### 2.2. Mapping Single Nucleotide Polymorphisms (SNPs)

**Figure 3:**
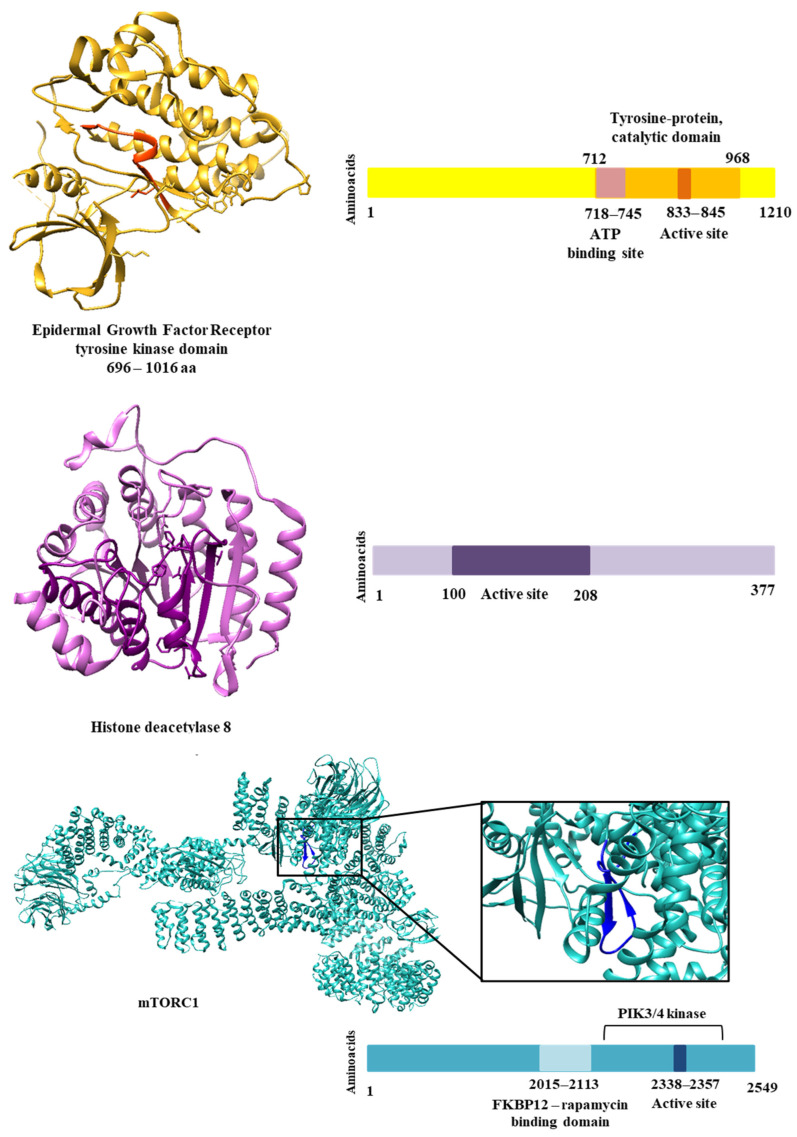

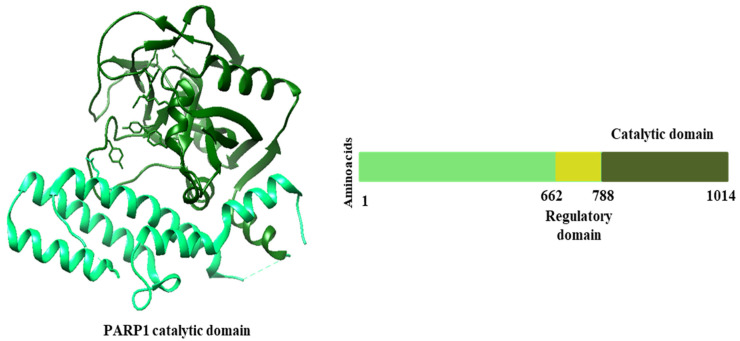
Three-dimensional structure of selected proteins. On the right side, information on domains and regions important for the biological activity of each protein.

Genomic analysis mapped nonsynonymous SNPs to regions of catalytic domains and the active site. Table 3 shows the clinically relevant SNPs that were selected for molecular docking studies. Figure 3 shows the regions of each protein that were analyzed. We identified 16 SNPs for the EGFR receptor, 28 SNPs for the HDAC8 protein, 40 SNPs for mTOR1 and 113 SNPs for the PARP1 protein. The choice of SNPs to design polymorphic mutations and undergo molecular docking was based on clinical relevance. Therefore, we considered the SNPS that presented some study related to an altered phenotype or disease. In addition, we selected SNPs with allele frequencies of the polymorphic allele greater than expected for certain populations. Therefore, we could select 4 SNPs for the EGFR receptor, 11 for the HDAC8 protein, 11 for mTOR1 and 2 for PARP1 for these analyses. Although the results show few SNPs with clinical relevance, most show a high probability that the polymorphic variant will affect protein function if the mutation occurs. This result was provided by Polyphen, a tool that predicts variants likely to affect protein function based on physical and comparative considerations.

### 2.3. Molecular Docking

The lignans with the highest values of predicted biological activity for EFGR, HDAC8, mTOR and PARP1 proteins were subjected to molecular docking. Docking was performed with the ancestral protein and the mutated proteins. Only one lignan (colocasinol A) was coupled to the EGFR receptor. The remaining lignans were renamed to better express the docking results in Table 4. For HDAC8, the lignans longipedlignan H, longipedlignan I and sinolignan A were renamed as lignan 1, lignan 2 and lignan 3, respectively. For mTOR, the lignans (1R,2R)-2-{4-[(1R,3aS,4R,6aS)-4-(4-hydroxy-3,5-dimethoxyphenyl)-hexahydrofuro[3,4-c] furan-1-yl]-2,6-dimethoxyphenoxy, bizanthplanispine B and bigraminol A were renamed as lignan 1, lignan 2 and lignan 3, respectively. Similar renaming was carried out with the lignans active against the PARP1 protein (longipedlignan H, longipedlignan I and longipedlignan J). The results were generated using the Moldockscore function. More negative values indicated better predictions. In this study, we only sought to analyze the difference in binding affinity according to the polymorphic variant. Thus, it is possible to verify the binding affinity contribution of a compound according to the genetic profile.

The docking results can be seen in Table 4. According to the results, the colocasinol A lignan presented similar values, except for the V845L variant, which obtained a binding energy value of −34.46 kcal/mol. For the HDAC8 protein, we observed that lignan 1 (longipedlignan H) failed to interact with the enzyme, despite the QSAR modeling results. The other lignans presented varied results, with more negative values for the ancestor and some variants, such as S199T. More negative values may indicate better binding affinity probability, suggesting stronger protein inhibition. We were unable to design the mutation in two variants for mTOR due to the incomplete ancestral protein available in databases. The results for mTOR revealed quite varied energy values when compared with the results for the ancestral protein. Only bizanthplanispine B obtained higher binding energy values than the ancestor. The lowest binding energy value for this protein was −34.21 kcal/mol for the M2327I variant. However, the bizanthplanispine B lignan obtained a high binding energy value (−96.85 kcal/mol) for this variant. These results show that not only the type of mutation interferes with binding affinity, but also the structural variety of compounds. Lastly, the PARP1 protein showed similar values, except for longipedlignan H, which did not interact with the protein.

**Figure 4:**
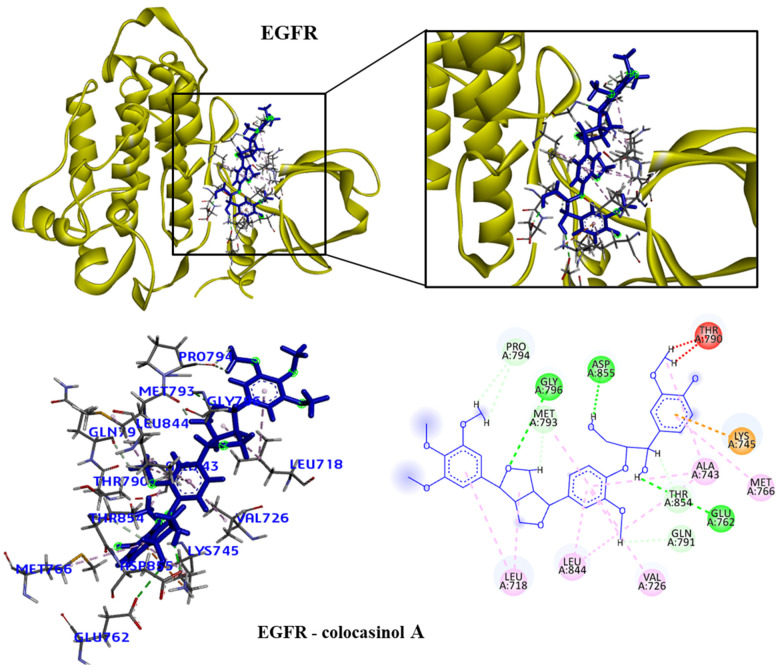
3D and 2D interactions of the EGFR receptor (4G5J) and the colocasinol A lignan. Hydrogen bonds are highlighted in dark green, van der Waals interactions are highlighted in light green, hydrophobic interactions are highlighted in pink and steric interactions are highlighted in red.

**Figure 5:**
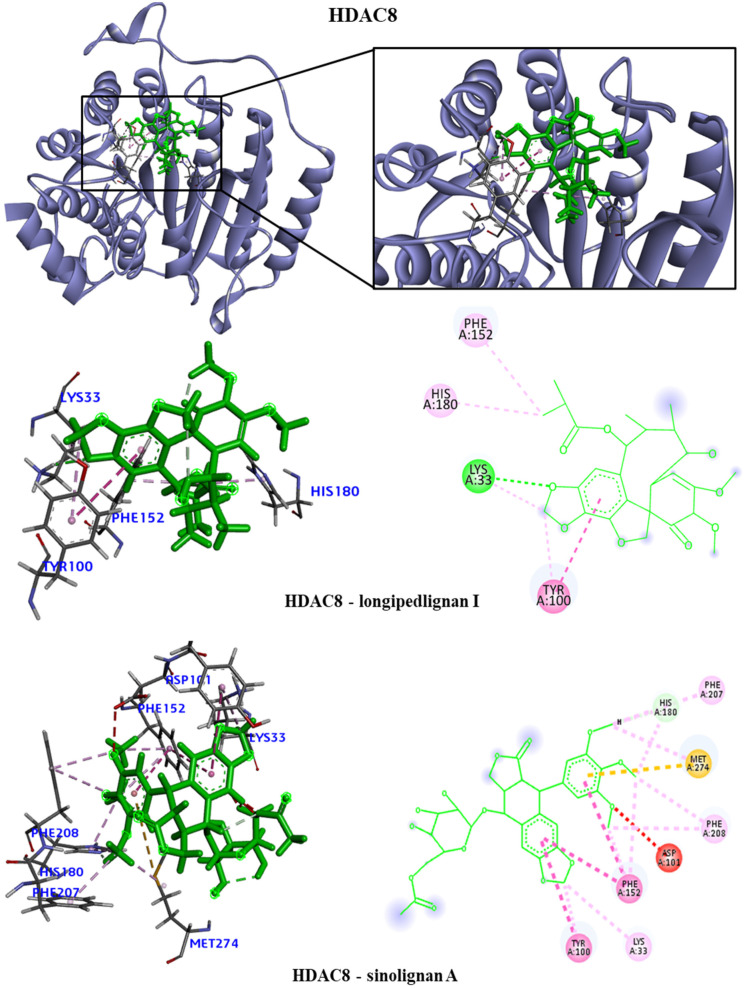
3D and 2D interactions of HDAC8 (1T67) with selected lignans. Hydrogen bonds are highlighted in dark green, van der Waals interactions are highlighted in light green, hydrophobic interactions are highlighted in pink and steric interactions are highlighted in red.

**Figure 6:**
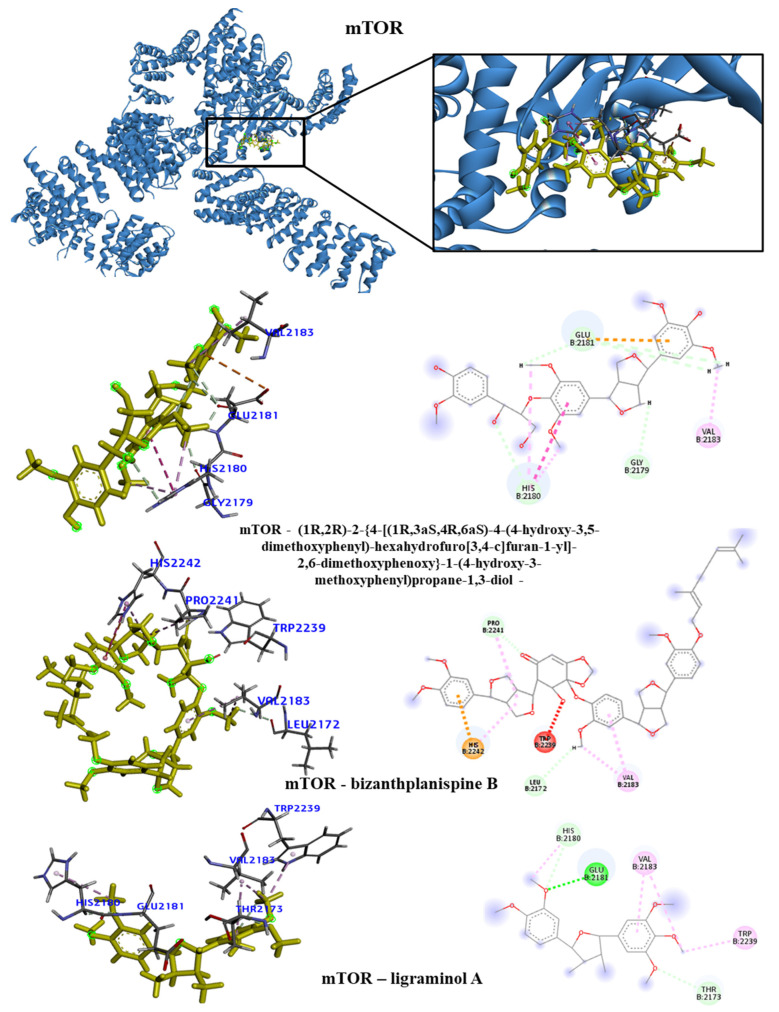
3D and 2D interactions of mTOR (7OWG) with selected lignans. Hydrogen bonds are highlighted in dark green, van der Waals interactions are highlighted in light green, hydrophobic interactions are highlighted in pink and steric interactions are highlighted in red.

**Figure 7:**
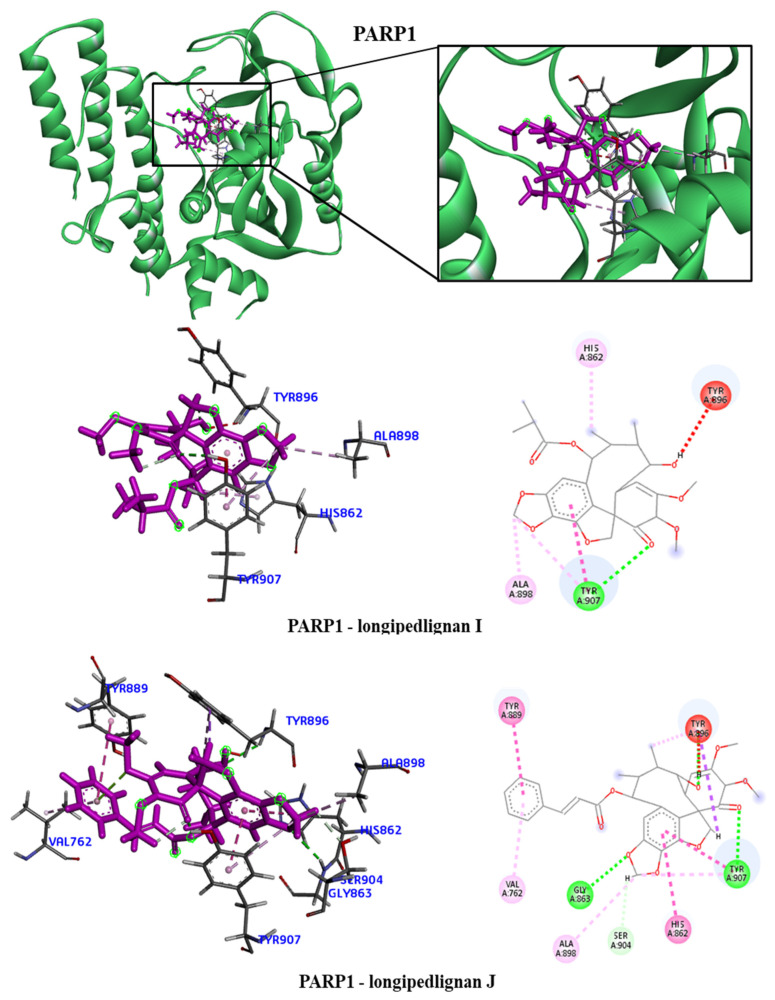
3D and 2D interactions of PARP1 (5A00) with selected lignans. Hydrogen bonds are highlighted in dark green, van der Waals interactions are highlighted in light green, hydrophobic interactions are highlighted in pink and steric interactions are highlighted in red.

We took the opportunity to analyze the observed interactions between the selected lignans and the proteins with the ancestral allele (Figure 4, Figure 5, Figure 6 and Figure 7). We observed that the EGFR receptor formed several hydrogen bonds, stabilizing the bonds with the Glu762, Glu796 and Asp855 amino acids, in addition to several hydrophobic interactions, including Leu718, Val726, Ala743, Met766 and Leu844. There was no interaction with the longipedlignan H lignan for the HDAC8 protein. However, interactions with the other lignans were observed, and we identified four common links/interactions between these compounds and the active site. The interactions were observed with Lys33, Tyr100, Phe152 and His180 residues. Moreover, three common interactions between lignans and the active site were observed for the mTOR protein.

The hydrophobic interactions formed were with the His2180, Glu2181 and Val2183 residues. The longipedlignan H lignan also did not interact with the active site for the PARP1 protein. Nevertheless, we analyzed the interactions with longipedlignan I and longipedlignan J lignans and observed interactions between them and the active site, forming interactions with the His862, Tyr896, Ala898 and Tyr907 amino acids.

### 2.4. Prediction of Absorption, Distribution, Metabolism, Excretion and Toxicity (ADMET) Properties

Many drugs used to treat cancer are toxic and have a variety of side effects. Therefore, in this study, we sought to verify the ADMET properties of lignans, with an emphasis on toxicity. The pharmacokinetic mechanisms that involve the steps of absorption, distribution, metabolism, excretion and toxicity (ADMET) when failures are found are considered the main causes of failure in developing drugs derived from natural or synthetic products. Therefore, virtual platforms such as pkCSM can be used to identify these possible problems through an in silico approach involving predictive models [25]. Thus, ADMET analyzes for the studied lignans were performed with the parameters shown in the tables below (Table 5). The colocasinol A, longipedlignan H, longipedlignan I, longipedlignan J, sinolignan A, (1R,2R)-2-{4-[(1R,3aS,4R,6aS)-4-(4-hydroxy-3,5-dimethoxyphenyl)-hexahydrofuro[3,4-c]furan-1-yl]-2,6-dimethoxyphenoxy, bizanthplanispine B and ligraminol lignans were renamed in these analyzes as L1, L2, L3, L4, L5 and L6, respectively.

Next, virtual models were used to evaluate the absorption parameters against Caco-2 cellular models, intestinal absorption and skin permeability. Caco-2 cell lines are composed of human epithelial colorectal adenocarcinoma cells widely used to predict oral drug absorption. In this predictive model, values above 0.90 are considered to demonstrate that the compounds have high permeation in these cells. Therefore, the lignans (L1, L2, L3 and L8) present satisfactory values. All lignans showed adequate values regarding the complementary result of intestinal absorption (the main drug absorption site), ranging from 89–100% absorption. Finally, all lignans showed (log Kp > −2.5) permeation values, suggesting low potential for transdermal administration [26].

It can be seen that lignans (L4, L5 and L6) are potential substrates with regard to possible interactions with P-glycoproteins (an important efflux pump that can compromise the bioavailability of xenobiotics). The modulation of P-glycoprotein-mediated transport has significant pharmacokinetic implications for the PgP substrate, which can be exploited for therapeutic advantages or result in contraindications in case there are interactions between drugs used concurrently with lignans [27].

Among the distribution parameters, it was possible to evaluate the theoretical volume of distribution (VDss)—the total dose of the drug distributed in a way that guarantees the same plasma concentration, permeability in the blood-brain barrier and permeation in the CNS. In view of this, the results obtained suggest that lignans (L3, L4 and L7) have a high volume of distribution with values (logL/kg > 0.45); however, all compounds have a low permeation potential in the brain with regard to bioavailability in the CNS (logBB < −1 and logPS < −3). Therefore, these results demonstrate that lignans have low pharmacological potential against neurological disorders and little influence on neurotoxic processes [28]. The in silico tool also made it possible to identify whether the structures are substrates or inhibitors of the mitochondrial enzymatic metabolism system—CYP450. This system constitutes an important family of monooxygenase capable of biotransforming 90% of xenobiotics, for which CYPA4, CYPB6, CYP3C19 and CYP2D6 stand out as being the main ones involved in the metabolic process [29]. In the table, it was possible to identify which isoforms are substrates and/or inhibitors of the lignans used in the study, emphasizing the importance of possible pharmacokinetic interactions of possible drugs which may act as enzyme inducers or inhibitors. Finally, the behavior of lignans with regard to excretion vis-à-vis organic cation transporters 2—an important renal uptake protein in the clearance process, and a point subject to pharmacokinetic interactions between drugs—was also predicted. As observed, lignans are not substrates for the transporter, in addition to presenting satisfactory renal clearance (proportionality constant (clot by the combination of hepatic and renal clearance)), which demonstrates good elimination capacity [30].

The preclinical safety profile is one of the main concerns in the development of new drugs that should present preserved efficacy and good topical and systemic tolerability. In assessing toxicity through virtual approaches, the toxicological profile of lignans was evaluated against the mutagenic potential in bacteria (AMES), potassium channels encoded by hERG, hepatotoxicity and skin sensitization. Based on the results obtained, it was seen that lignans do not present toxicity according to the AME model, do not inhibit hERG I channels and do not promote skin sensitization. However, L5, L6 and L7 can inhibit hERG II. This condition may be related to echocardiographic changes called acquired long QT syndrome, which promotes ventricular arrhythmias [27]. Therefore, it is important to point out that the pharmacokinetic determination may substantially contribute to the preclinical research of these derivatives and early recognition of possible ADMET failures.

## 3. Discussion

We found no structural correlation between subclasses of lignans and anticancer activity in the literature. This is the first work that correlates the structure of lignan subclasses with anticancer activity. Among the ten subclasses of lignans, three were considered the most promising for anticancer activity: furofurans, dibenzocyclooctadiens and aryltretalins. This result was based on the amount of lignans active against antitumor activity for each subclass. The pActivity values were approximated between lignans but are significant when greater than 0.50. These values refer to the prediction of inhibition based on the data of biological activities obtained for the construction of the models.

Furofuran lignans contain the backbone of 2,6-diaryl-3,7-dioxabicyclo[3.3.0]octane and represent one of the major subclasses of the lignan family. Furofuran lignans have a wide variety of structures due to different substituents on the aryl groups and diverse configurations on the furofuran ring. Antioxidant, anti-inflammatory, cytotoxic and antimicrobial activities are among the main activities reported for furofuran lignans [23]. Our study contributes to further explore furofuran lignans as anticancer agents.

One example is a study by Cheng et al. [31], who isolated five new furofuran lignans, brasesquilignan A-E (1–5), from *Selaginella braunii* Baker. All of these compounds were evaluated for antiproliferative activities against various human cancer cells in vitro but showed weak inhibition. In addition, Vitória et al. [32] isolated two new tetrahydrofuran lignans, taungtangyiols A and B, and eight known furofuran lignans from *Premna integrifolia* wood. Taungtangyiols A and B were observed to inhibit melanin deposition in B16F10 mouse melanoma cells, with IC_50_ values of 50.7 and 40.9 μM, respectively, without notable cytotoxicity.

Another study isolated seven dibenzocyclooctadiene lignans from the fruits of *Schisandra chinensis*. Cell viability assays verified the cytotoxicity of isolated dibenzocyclooctadiene lignans against AGS, HeLa and HT-29 human cancer cells [33].

Podophyllotoxin (PTOX, 1) is an aryltetralin-type lignan, first discovered in the plant *Podophyllum peltatum*. It has been used in biosynthesis and total synthesis as a prospective alternative due to its potent anticancer activities [34,35]. Another lignan structurally and closely related to podophyllotoxin is deoxypodophyllotoxin. This aryltetralin is a potent antitumor and anti-inflammatory agent and is especially used as a precursor for the semi-synthesis of etoposide phosphate and teniposide cytostatic drugs. These analogues are also used in cancer therapy [36].

Another study by Zilla et al. [37] extracted six aryltetralin-type lignans from the root of *Podophyllum hexandrum* as a potential source of bioactive lead metabolites with anticancer activity. Aryltetralins are designated as 4′-demethyl-deoxypodophyllotoxin, podophyllotoxin, 4′-demethyl-podophyllotoxin, podophyllotoxin-4-O-β-d-glucopyranoside, 4′-demethyl-deoxypodophyllotoxin-4-O-β-d-glycopyranoside and 4′-demethyl-podophyllotoxin-4-O-β-d-glucopyranoside. All aryltetralins exhibited remarkable cytotoxic potential in several cancer cell lines. The latter was observed to increase apoptotic cascades in MCF-7 breast cancer cells (i.e., nuclear condensation, membrane blebbing), probably by destabilizing the microtubular protein tubulin. It additionally binds and modulates checkpoint kinase-2, a key cell cycle regulatory protein in normal and cancer cells.

Correlation studies between the chemical structure and anticancer activities are important to reduce costs in identifying new drugs and to develop more potent drugs. An example of this is the study carried out by Scotti et al. [38], who tabulated the most important examples of determined virtual screening for anticancer flavonoids and highlighted the structural determinants. Like lignans, flavanoids have a structural variety and different described biological activities. The aforementioned study identified the action mode, the most potent anticancer flavonoids and tips for the structural design of anticancer flavonoids in a review.

According to Silva and Alcorn (2019), the complexities in tumor heterogeneity and interconnections between various pathways led researchers to use the multidirected approach of broad spectrum. The authors cite various targets of the lignans, including Cyclin-dependent Kinase 2 (CDK2), Proliferating Cell Nuclear Antigen (PCNA), Platelet-Derived Growth Factor (PDGF), Akt Serine/Threonine Kinase 1 (AKT1), insulin-bike Growth Growth Factor 1 receptor (IGFR), EGFR, Cyclogenase 1/2 (COX1/2), Breast Cancer 1 (BRCA1), Vascular Endothelial Growth Factor (VEGF), P53, Caspase 3 and B-Cell Lymphoma 2 (BCL-2), among others. Therefore, our study also contributes to the reporting of new targets for the development of antitumor drugs.

## 4. Materials and Methods

### 4.1. Data Collection and Curation

This study selected and investigated five proteins involved in the pathogenesis of cancer with sufficient and available biological activity and involved in the development of different types of cancer in different pathways. Compounds with known biological activity for ABL, EGFR, HDAC, mTOR and PARP were obtained (https://www.ebi.ac.uk/chembl/ (accessed on 14 February 2023)) [39,40]. Details of the pools can be found in Table 6. Compounds were ranked based on pIC_50_ [−log IC_50_ (mol/L)]. The IC_50_ value represents the concentration required for 50% inhibition. The compounds and these data were used to build predictive models with biological activity for the indicated proteins. In addition, 495 lignan subclasses were evaluated by ligand-based virtual screening to identify molecules with potential activity against the selected proteins. The lignan subclasses are formed by (35) Dibenzylbutanes, (12) 3,4-Dibenzyltetrafurans, (30) Dibenzylbutyrolactones, (147) Dibenzocyclooctadienes, (72) Aryltetralins, (17) Arylhydronaphthalene, (25) Arylnaphthalenes, (66) Furofuran, (37) 2,5-diarlytetrahydrofuran and (54) 2-aryl-4-benzyltetrahydrofuran. The three-dimensional structures were generated by Chemaxon Standardiser v.18.17.0, (www.chemaxon.org (accessed on 20 February 2023)).

### 4.2. QSAR Modelling

Compounds with known biological activity towards the proteins ABL, EGFR, HDAC, mTOR and PARP were saved in special data file format (SDF) and imported into the Dragon 7.0 program [41] to generate descriptors. A predictive model was built for each protein bank. The descriptors referring to each bank and biological activity information were imported into the Knime 3.5.3 program (KNIME 3.5.3, Konstanz Information Miner Copyright, 2018, www.knime.org (accessed on 22 February 2023)) to generate the predictive models. The data were divided in a “Partitioning” tool using the “Stratified sample” option in the program, which separated the data into training and test sets representing 80% and 20% of all compounds, respectively. The sets were randomly selected, but the proportions of active and inactive substances were maintained in both databases. We used the random forest (RF) algorithm to build predictive models. Cross-validation was performed to estimate the predictive power of the developed models.

The external performances of the selected models were analyzed for sensitivity, specificity and accuracy. In addition, receiver operating character (ROC) curve sensitivity and specificity were used because they describe actual performance more clearly than accuracy. The Matthews correlation coefficient (MCC) was used to evaluate the model overall based on the results obtained in the confusion matrix [42]. The applicability domain (APD) was used to analyze the compounds in the test sets, if the predictions are reliable [43,44]. The lignans considered active against the selected proteins were submitted to the other methodologies.

### 4.3. Mapping SNPs

Identifying single nucleotide polymorphisms (SNPs) in important regions of proteins involved in the progression of different types of tumors can help rational drug design. In addition, it contributes to developing new therapies based on the genetic profile. Genetic variations in target proteins were identified by searching two databases. The National Center for Biotechnology Information (https://www.ncbi.nlm.nih.gov/ (accessed on 8 March 2023)) and Ensembl (https://www.ensembl.org/index.html (accessed on 8 March 2023)) databases [45] were consulted to obtain essential information about genes, phenotype and SNPs in the investigated proteins. SNPs with varied allele frequencies in catalytic domain and active site regions were considered in the study. Mutations with the polymorphic variants were designed using the UCSF Chimera program (Visualization and Informatics—RBVI, San Francisco, CA, USA) [46].

### 4.4. Molecular Docking

Molecular docking was performed using Molegro Virtual Docker v6.0.1 (MVD) program (Molexus IVS Rørth Ellevej 3, Odder, Denmark) [47] on the five targets selected for anchoring studies (Table 7). The MolDock score algorithm was used as a scoring function to predict the best interactions between ligand and receptor. The 3D structures of the proteins used in this study were obtained from the Protein Data Bank (PDB) [48,49] (Table 7). All water molecules were initially removed from the crystalline structure and the root-mean-square deviation (RMSD) was calculated from the poses to indicate the degree of reliability of the fit. RMSD values less than 2.0 Å were considered successful.

### 4.5. Prediction of ADMET Properties

ADMET parameters were calculated using the open access web tool SwissADME (http://www.swissadme.ch (accessed on 18 February 2023)) [50] and ADMET profiles of lignans were investigated using the pkCSM web platform (https://biosig.lab.uq.edu.au/pkcsm/ (accessed on 18 February 2023)) [26], which offers a set of rapid predictive models to evaluate physicochemical, pharmacokinetic, pharmacological and toxicity properties.

## 5. Conclusions

It is not easy to correlate anticancer activity with a particular subclass due to the structural diversity of lignans and the various pharmacological properties. Therefore, the present study used a set of lignans distributed in the 10 known subclasses and submitted them to in silico methodologies to verify the anticancer potential of these compounds. Five predictive models were built against important targets in cancer development. The RF model was able to select a potentially active lignan against the EFGR receptor, 86 lignans considered active against HDAC, 155 lignans active against the mTOR protein and 156 lignans active against the PARP protein. Overall, the predictions of biological activities showed inhibition values ranging from 50–63%. No compound was active against the ABL protein. The results also showed that the subclasses with the most active compounds were dibenzocyclooctadiene, furofuran and aryltetralin for all proteins. These subclasses are interesting for anticancer activity. The other subclasses are correlated with other pharmacological properties known for lignans, such as anti-inflammatory, antioxidant and trypanocidal activity. The lignans considered most active for each protein (colocasinol A for EGFR; longipedlignan H, longipedlignan I and sinolignan A for HDAC8; (1R,2R)-2-{4-[(1R,3aS,4R,6aS)-4-(4-hydroxy-3,5-dimethoxyphenyl)-hexahydrofuro[3,4-c]furan-1-yl]-2,6-dimethoxyphenoxy, bizanthplanispine B and ligraminol A for mTOR and longipedlignan H, longipedlignan I and longipedlignan J for PARP1) were subjected to molecular docking.

Genomic analysis selected 4 clinically relevant SNPs for the EGFR receptor, 11 for the HDAC8 protein, 11 for mTOR1 and 2 for PARP1. The projected mutations and molecular docking results showed differences in binding energy values between proteins and between selected lignans. mTOR presented the most discrepant energy values. From these results, we suggest that mutations in the catalytic domain of target proteins can generate strong or low inhibition depending on the type of variant present in the individual.

We also suggest with this study exploit the use of lignans analogs in order to generate preliminary data from the structure–activity relationship (SAR), which could provide more information on the relationship between structural modifications and the anticancer activity of lignans.

## Figures and Tables

**Table 1:** Performance summary corresponding to the results obtained for all random forest models.

Enzyme	Validation	Accuracy	Sensitivity	Specificity	PPV *	NPV *	MCC	ROC
**ABL**	Test	0.99	1	0.99	0.99	1	0.76	0.95
	Cross	0.88	0.86	0.90	0.89	0.86	0.82	0.94
**EFGR**	Test	0.82	0.84	0.80	0.82	0.82	0.65	0.90
	Cross	0.83	0.86	0.81	0.83	0.84	0.67	0.91
**HDAC**	Test	0.82	0.80	0.84	0.83	0.80	0.64	0.91
	Cross	0.81	0.84	0.79	0.80	0.83	0.63	0.89
**mTOR**	Test	0.85	0.89	0.80	0.85	0.85	0.70	0.93
	Cross	0.84	0.88	0.79	0.84	0.84	0.68	0.92
**PARP**	Test	0.86	0.82	0.84	0.85	0.87	0.72	0.92
	Cross	0.83	0.85	0.81	0.81	0.84	0.66	0.90

* PPV—positive predicted value. * NPV—negative predicted value.

**Table 2:** Number of active lignans divided by subclass and the respective probability of biological activity for each enzyme.

Protein	Subclass	Active Compounds	pActivity
**EFGR**	Furofuran	**1**	0.54
**HDAC**	Dibenzilbutirolactone	**12**	0.50–0.55
	Dibenzociclooctadiene	**22**	0.50–0.63
	Aryltetralin	**16**	0.50–0.63
	Arylhydronafthalene	**4**	0.50
	Arylnaftalene	**2**	0.50–0.52
	Furofuran	**22**	0.50–0.57
	2-aryl-4-benzyltetrahidrofuran	**8**	0.51–0.56
**mTOR**	Dibenzylbutane	**3**	0.50–0.53
	Dibenziltetrafuran	**2**	0.50–0.56
	Dibenzilbutirolactone	**14**	0.50–0.56
	Dibenzociclooctadiene	**40**	0.50–0.55
	Aryltetralin	**28**	0.50–0.56
	Arylhydronafthalene	**6**	0.50–0.54
	Arylnaftalene	**8**	0.50–0.56
	Furofuran	**37**	0.50–0.58
	2,5-diarlytetrahydrofuran	**3**	0.50–0.58
	2-aryl-4-benzyltetrahidrofuran	**14**	0.50–0.58
**PARP**	Dibenzylbutane	**1**	0.50
	Dibenzilbutirolactone	**2**	0.52–0.54
	Dibenzociclooctadiene	**89**	0.50–0.61
	Aryltetralin	**18**	0.50–0.54
	Arylhydronafthalene	**4**	0.50–0.51
	Arylnaftalene	**8**	0.53–0.56
	Furofuran	**30**	0.50–0.56
	2-aryl-4-benzyltetrahidrofuran	**4**	0.52–0.55

**Table 3:** Clinically relevant SNPs located in the catalytic domain selected for molecular docking analyses.

Enzyme	Aminoacid	SNP	Alleles	Ancestral Amino Acid	Polymorphic Amino Acid	Ancestor Allele Frequency	Allelic Frequency of the Polymorphism	Polyphen
**EGFR**	833	rs397517126	T/G	L	V	-	-	0.829
	835	rs397517128	A/T	H	L	-	-	0.999
	842	rs1003269794	A/C/G	N	H/D	-	-	1
	845	rs1787407031	G/C	V	L	-	-	0.428
**HDAC8**	101	rs2051867176	T/C	D	G	-	-	1
	139	rs878853048	C/G	G	A	-	-	0.999
	140	rs1569412360	C/T	G	R	-	-	1
	140	rs1057518047	C/A	G	V	-	-	1
	145	rs2051860492	T/C	K	E	-	-	0.727
	155	rs2048985556	G/A	L	F	-	-	0.017
	176	rs1057518727	T/C	D	G	-	-	1
	186	rs797045612	C/T	E	K	-	-	0.923
	188	rs1603069440	C/T	A	T	-	-	0.997
	195	rs1556009247	A/C/T	V	G/D	-	-	1
	199	rs1057518126	A/T	S	T	-	-	0.979
**mTOR1**	2326	rs1642201364	C/A	V	F	-	-	
	2327	rs878855328	C/T	M	I	-	-	
	2367	rs1642080627	T/C	T	A	-	-	
	2406	rs1557739557	C/A/T	V	L/M	-	-	
	2413	rs1553171141	C/A	S	I	-	-	
	2416	rs1173643064	G/A	A	V	-	-	
	2419	rs587777900	C/T	E	K	-	-	
	2427	rs1085307113	A/G/T	L	P/Q	-	-	
	2431	rs1057524044	A/G	L	P	-	-	
	2457	rs1060501911	A/G	I	T	-	-	
	2458	rs1641759287	C/A	L	F	-	-	
**PARP1**	864	rs993561075	A/C	S	A	0.5	0.5	0.993
	940	rs3219145	T/C/G	K	R/T	0.998329	0.001671	0.79

**Table 4:** Binding energy values obtained from molecular docking for lignans with higher biological activity prediction values. Results with higher binding energy values are highlighted in bold.

Enzyme	Ancestor/Mutation	Binding Energy Values		
		Lignan 1	Lignan 2	Lignan 3
**EGFR**	Ancestor	−24.04		
	L833V	−20.91		
	H835L	−18.77		
	N842H	−23.86		
	V845L	−34.46		
**HDAC8**	Ancestor	-	−91.43	−72.66
	D101G	-	−76.40	−103.84
	G139A	-	−84.29	−75.52
	G140R	-	−85.82	−95.60
	G140V	-	−80.36	−77.22
	K145E	-	−83.07	−76.75
	L155F	-	−84.68	−104.49
	D176G	-	−76.04	−109.54
	E186K	-	−77.42	−74.81
	A188T	-	−87.21	−92.01
	V195G	-	−75.94	−90.33
	S199T	-	−97.80	−102.33
**mTOR**	Ancestor	−84.51	−57.71	−85.76
	V2326F	−56.35	−92.26	−45.96
	M2327I	−34.21	−96.85	−49.40
	T2367A	−49.53	−78.14	−56.66
	V2406L	−69.68	−78.05	−51.91
	S2413I	−44.52	−90.24	−48.06
	E2419K	−69.90	−100.11	−47.54
	L2427P	−63.85	−63.74	−39.79
	L2431P	−64.20	−85.28	−43.77
**PARP1**	Ancestor	-	−136.95	−172.23
	S864A	-	−134.17	−157.52
	K940R	-	−137.38	−154.40

**Table 5:** ADMET properties of lignans selected by QSAR and molecular modeling studies.

Absorption	L1	L2	L3	L4	L5	L6	L7	L8
Caco2 permeability (log Papp in 10^−8^ cm/s)	1.325	1.214	1.272	0.337	0.597	0.496	0.555	1.249
Intestinal absorption (% absorbed)	89.098	100	100	100	53.666	67.479	100	97.43
Skin permeability (log Kp)	−2.747	−2.736	−2.735	−2.735	−2.735	−2.735	−2.735	−2.82
P-glycoprotein substrate	No	No	No	Yes	Yes	Yes	No	No
**Distribution**	**L1**	**L2**	**L3**	**L4**	**L5**	**L6**	**L7**	**L8**
VDss (log L/kg)	0.064	−0.285	−0.714	−0.484	0.138	−0.101	−0.496	−0.052
BBB permeability (log BB)	−0.55	−1.189	−1.19	−1.899	−1.941	−1.848	−2.389	−0.026
CNS permeability (log PS)	−2.966	−2.846	−2.904	−4.136	−3.799	−3.674	−3.219	−2.613
**Metabolism**	**L1**	**L2**	**L3**	**L4**	**L5**	**L6**	**L7**	**L8**
CYP2D6 (substrate)	No	No	No	No	No	No	No	No
CYP3A4 (substrate)	No	Yes	Yes	Yes	Yes	Yes	Yes	Yes
CYP1A2 (inhibitor)	No	No	No	No	No	No	No	No
CYP2C19 (inhibitor)	No	No	No	No	No	No	No	Yes
CYP2C9 (inhibitor)	No	No	No	No	No	Yes	No	Yes
CYP2D6 (inhibitor)	No	No	No	No	No	No	No	No
CYP3A4 (inhibitor)	No	No	Yes	No	No	Yes	No	Yes
**Excretion**	**L1**	**L2**	**L3**	**L4**	**L5**	**L6**	**L7**	**L8**
Total clearance (log ml/min/Kg)	0.171	0.293	0.225	0.156	0.192	0.259	−0.498	0.132
Renal OCT2 (substrate)	No	No	No	No	No	No	No	No
**Toxicity**	**L1**	**L2**	**L3**	**L4**	**L5**	**L6**	**L7**	**L8**
AMES toxicity	No	No	No	No	No	No	No	No
hERG 1 inhibitor	No	No	No	No	No	No	No	No
hERG 2 inhibitor	No	No	No	No	Yes	Yes	Yes	No
Hepatotoxicity	No	Yes	No	No	No	No	No	No
Skin sensitization	No	No	No	No	No	No	No	No

**Table 6:** Set of molecules from the ChEMBL databases for each enzyme selected in the study.

Database	ID ChEMBL	Active Molecules	Inactive Molecules	Total
**ABL**	CHEMBL1862	793 (pIC_50_ ≥ 7.0)	802 (pIC_50_ < 7.0)	1595
**EGFR**	CHEMBL203	3756 (pIC_50_ ≥ 6.0)	3464 (pIC_50_ < 6.0)	7220
**HDAC**	CHEMBL2093865	968 (pIC_50_ ≥ 6.3)	941 (pIC_50_ ≥ 6.3)	1909
**mTOR**	CHEMBL2842	2235 (pIC_50_ ≥ 7.0)	1753 (pIC_50_ ≥ 7.0)	3988
**PARP**	CHEMBL3105	977 (pIC_50_ ≥ 7.0)	982 (pIC_50_ ≥ 7.0)	1959

**Table 7:** Information (including RMSD) about the selected proteins, obtained from the PDB database and used for docking.

PDB ID	Protein	Class	RMSD	PDB Ligand	Resolution
**4G5J**	EGFR	Transferase	0.20	Afatinibe	2.80 Å
**1T67**	HDAC8	Hydrolase	7.46	B3N	2.31 Å
**7OWG**	mTOR	Signaling protein	-	-	4.70 Å
**5A00**	PARP1	Transferase	0.38	4.70 Å	2.75 Å

## Data Availability

Not applicable.

## Supplementary Materials

The following supporting information can be downloaded at: https://www.mdpi.com/article/10.3390/molecules28166011/s1, Figure S1: ROC (Receiver Operating Characteristic) curve generated for the five selected protein models. (**a**) ABL test; (**b**) ABL cross; (**c**) EFGR test; (**d**) EFGR cross; (**e**) HDAC test; (**f**) HDAC cross; (**g**) mTOR test; (**h**) mTOR cross; (**i**) PARP test; and (**j**) PARP cross.

## Sample Availability

Not available.
