# Supplementary material for: Virtual Screening of Different Subclasses of Lignans with Anticancer Potential and Based on Genetic Profile
Source: Molecules. 2023 Aug 11;28(16):6011. doi: 10.3390/molecules28166011 (PMC10459202; doi:10.3390/molecules28166011)
Supplement: Supplementary file 1 [file molecules-28-06011-s001.zip › molecules-2471996-supplementary.pdf]

Article

Virtual screening of different subclasses of lignans with anticancer potential and based on genetic profile

Mayara dos Santos Maia[1], Francisco Jaime Bezerra Mendonça-Junior [2,5] *, Gabriela Cristina Soares Rodrigues[3], Adriano Soares da Silva[4], Niara Isis Pereira de Oliveira[4], Pablo Rayff da Silva[5], Cícero Francisco Bezerra Felipe[5], Ana Pavla Almeida Diniz Gurgel[6], Anuraj

[1] Department of Molecular Biology, Federal University of Paraíba, João Pessoa-PB, Brazil; mayarasm@ltf.ufpb.br.
[2] Laboratory of Synthesis and Drug Delivery, State Universtiy of Paraiba, João Pessoa-PB, Brazil; franciscojaime@servidor.uepb.edu.br
[3] Miriri Foods and Bioenergy S/A; gaby.ecologia@gmail.com.
[4] Program in Ecology and Environmental Monitoring, Federal University of Paraíba, João Pessoa-PB, Brazil; Adriano.soares05071997@gmail.com; naira.isis.ni@gmail.com.
[5] Postgraduate Program in Natural Synthetic and Bioactive Products, Federal University of Paraíba, João Pessoa-PB, Brazil; pablo-rayff@hotmail.com; cicero@dbm.ufpb.br.
[6] Program in Cellular and Molecular Biology, Federal University of Paraíba, João Pessoa-PB, Brazil; ana.pavla@academico.ufpb.br.
[7] In Silico Research Laboratory, Eminent Bioscience, Indore, Madhya Pradesh, India; anuraj@eminentbio.com
[8] Laboratory of Cheminformatics, Program of Natural and Synthetic Bioactive Products (PgPNSB), Health Sciences Center, Federal University of Paraíba, João Pessoa-PB, Brazil; luciana.scotti@gmail.com; mtscotti@gmail.com.
* Correspondence: franciscojaime@servidor.uepb.edu.br

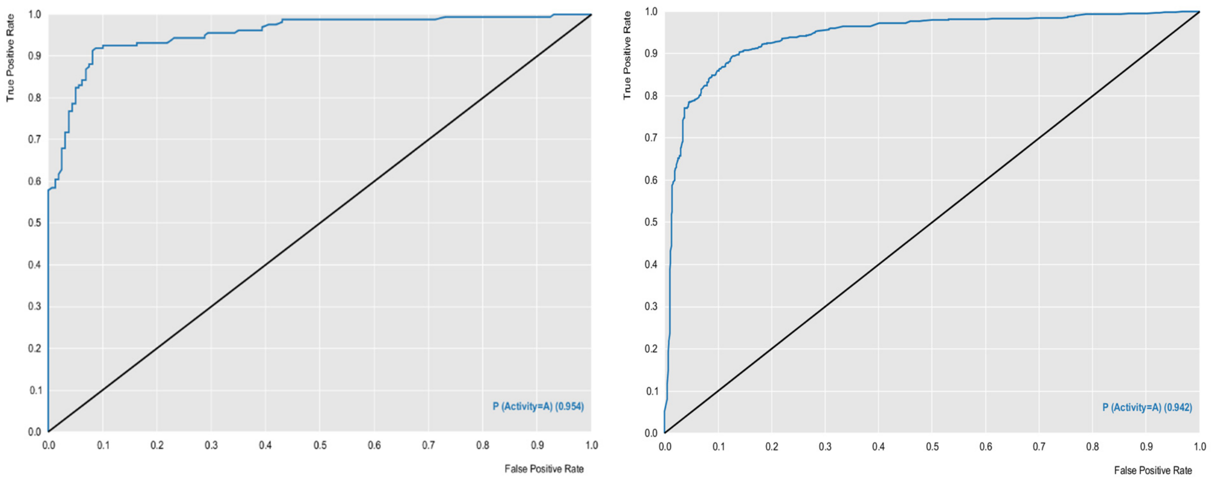

(**a**) (**b**)

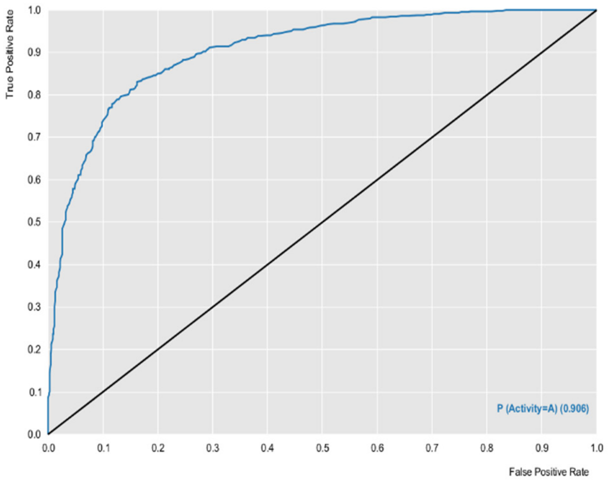
True Positive Rate
False Positive Rate
P (Activity=A) (0.906)

(c)

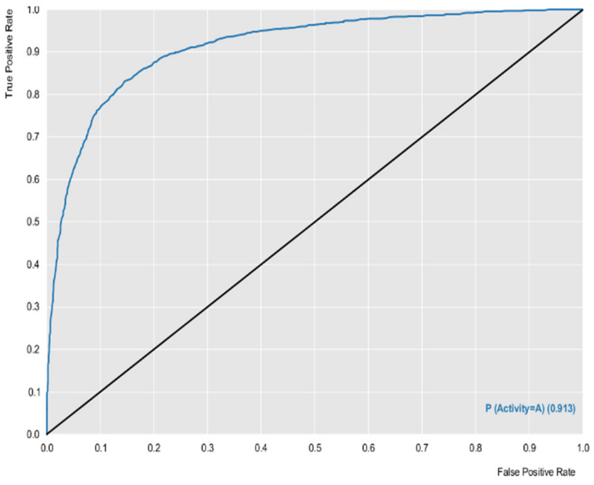
True Positive Rate
False Positive Rate
P (Activity=A) (0.913)

(d)

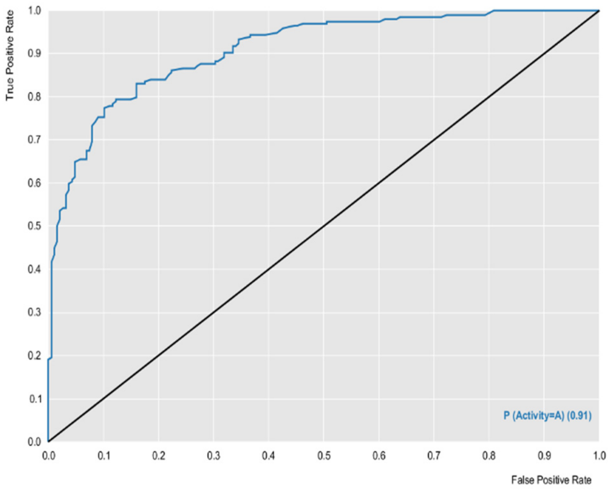
True Positive Rate
False Positive Rate
P (Activity=A) (0.91)

(e)

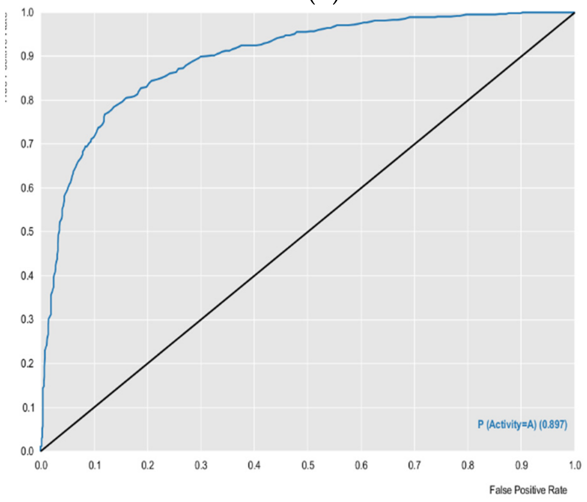
False Positive Rate
P (Activity=A) (0.897)

(f)

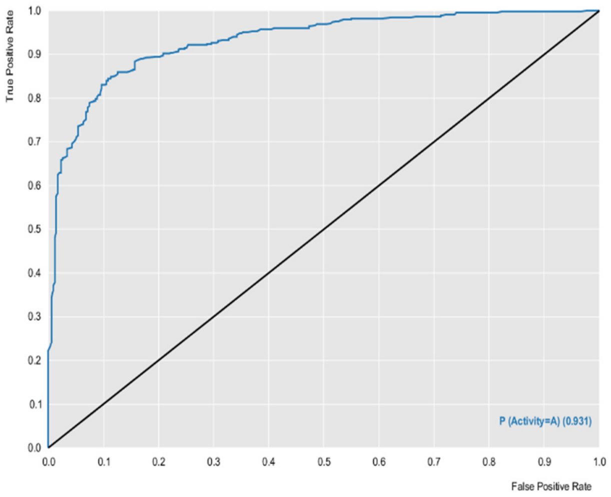
True Positive Rate
False Positive Rate
P (Activity=A) (0.931)

(g)

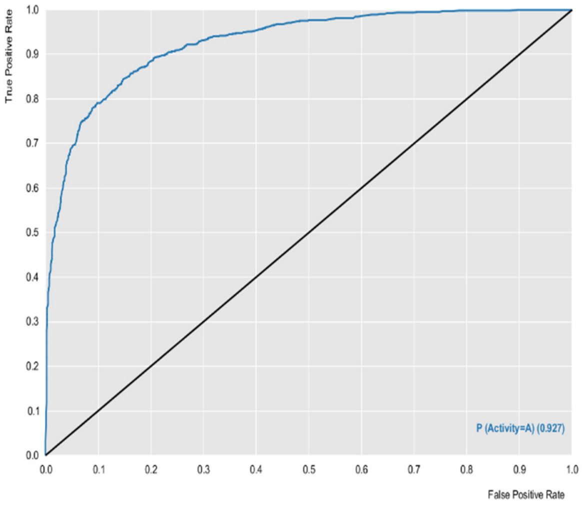
True Positive Rate
False Positive Rate
P (Activity=A) (0.927)

(h)

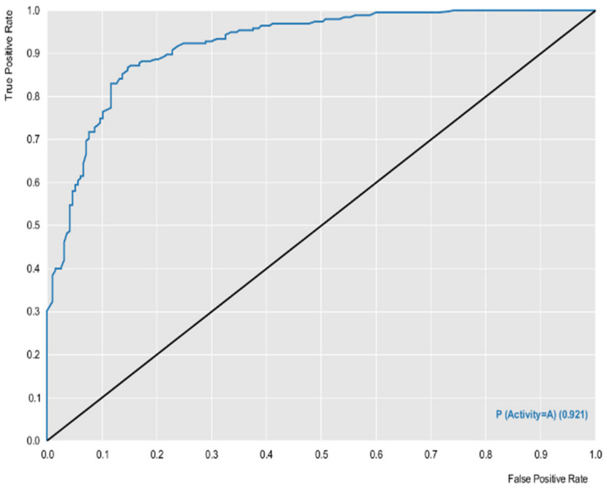

(**i**)

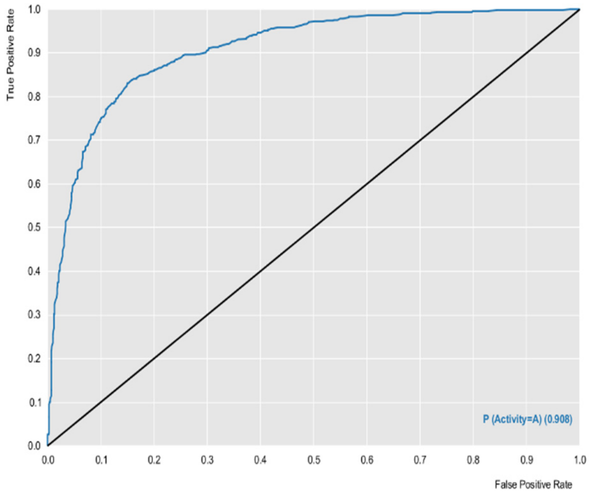

(**j**)

Figure 1. ROC (Receiver Operating Characteristic) curve generated for the five selected protein models. (**a**) ABL test; (**b**) ABL cross; (**c**) EFGR test; (**d**) EFGR cross; (**e**) HDAC test; (**f**) HDAC cross; (**g**) mTOR test; (**h**) mTOR cross; (**i**) PARP test; and (**j**) PARP cross.
